# Activity of the novel BCR kinase inhibitor IQS019 in preclinical models of B-cell non-Hodgkin lymphoma

**DOI:** 10.1186/s13045-017-0447-6

**Published:** 2017-03-31

**Authors:** P. Balsas, A. Esteve-Arenys, J. Roldán, L. Jiménez, V. Rodríguez, J. G. Valero, A. Chamorro-Jorganes, R. Puig de la Bellacasa, J. Teixidó, A. Matas-Céspedes, A. Moros, A. Martínez, E. Campo, A. Sáez-Borderías, J. I. Borrell, P. Pérez-Galán, D. Colomer, G. Roué

**Affiliations:** 1 Division of Hematology and Oncology, Institut d’Investigacions Biomèdiques August Pi iSunyer (IDIBAPS), Barcelona, Spain; 2 Grup d’Enginyeria Molecular, Institut Químic de Sarrià, Universitat Ramon Llull, Barcelona, Spain; 3 Department of Pathology, Hematopathology Unit, Hospital Clinic, Barcelona, Spain; 4 Pangaea Biotech S.L., Quiron Dexeus University Hospital, Barcelona, Spain

**Keywords:** B-NHL, Btk, Lyn, Syk, Cell migration, Mouse model

## Abstract

**Background:**

Pharmacological inhibition of B cell receptor (BCR) signaling has recently emerged as an effective approach in a wide range of B lymphoid neoplasms. However, despite promising clinical activity of the first Bruton’s kinase (Btk) and spleen tyrosine kinase (Syk) inhibitors, a small fraction of patients tend to develop progressive disease after initial response to these agents.

**Methods:**

We evaluated the antitumor activity of IQS019, a new BCR kinase inhibitor with increased affinity for Btk, Syk, and Lck/Yes novel tyrosine kinase (Lyn), in a set of 34 B lymphoid cell lines and primary cultures, including samples with acquired resistance to the first-in-class Btk inhibitor ibrutinib. Safety and efficacy of the compound were then evaluated in two xenograft mouse models of B cell lymphoma.

**Results:**

IQS019 simultaneously engaged a rapid and dose-dependent de-phosphorylation of both constitutive and IgM-activated Syk, Lyn, and Btk, leading to impaired cell proliferation, reduced CXCL12-dependent cell migration, and induction of caspase-dependent apoptosis. Accordingly, B cell lymphoma-bearing mice receiving IQS019 presented a reduced tumor outgrowth characterized by a decreased mitotic index and a lower infiltration of malignant cells in the spleen, in tight correlation with downregulation of phospho-Syk, phospho-Lyn, and phospho-Btk. More interestingly, IQS019 showed improved efficacy in vitro and in vivo when compared to the first-in-class Btk inhibitor ibrutinib, and was active in cells with acquired resistance to this latest.

**Conclusions:**

These results define IQS019 as a potential drug candidate for a variety of B lymphoid neoplasms, including cases with acquired resistance to current BCR-targeting therapies.

**Electronic supplementary material:**

The online version of this article (doi:10.1186/s13045-017-0447-6) contains supplementary material, which is available to authorized users.

## Background

The B cell receptor (BCR) regulates multiple cellular processes which are critical for maintenance and survival of B cells, including proliferation, differentiation, and cell migration [1]. Antigen engagement to BCR extracellular domain leads to phosphorylation and activation of immunoreceptor tyrosine-based activation motifs located in the cytoplasmic portion and other proteins downstream the receptor. Within BCR signalosome, the Lck/Yes novel tyrosine kinase (Lyn) recruits and phosphorylates the spleen tyrosine kinase (Syk), triggering a proliferation and survival cascade signaling that involves the phosphorylation and activation of Brutons’ tyrosine kinase (Btk), which subsequently phosphorylates phospholipase Cγ2 (PLCγ2), leading to calcium mobilization and activation of several downstream pathways, including MAP kinases, Akt and NF-κB [2]. In addition to tonic, ligand-mediated BCR signaling, chronic BCR activation can occur in the absence of antigen engagement [3], leading to aberrant, constitutive BCR activation in several B cell non-Hodgkin lymphoma (B-NHL) subtypes, including diffuse large B-cell lymphoma (DLBCL), mantle cell lymphoma (MCL), follicular lymphoma (FL), and chronic lymphocytic leukemia (CLL) [4–7]. In these entities, BCR signaling represents an important pro-survival stimulus that may be stronger than in normal B cells, supporting the recent emergence of several BCR-targeting therapies [7]. But despite the promising results obtained with the first kinase inhibitors, such as fostamatinib and ibrutinib, specific for the Src-family kinases Syk and Btk [8], the design of new compounds is warranted to improve treatment efficacy and to by-pass the resistance appearing in primarily responsive patients [9–11]. In this context, we recently described the synthesis of a new family of 4-aminopyrido[2,3-*d*]pyrimidines with kinase inhibitory property and antitumoral activity in B lymphoid cells. Compound 19 (thereafter referred as IQS019) was identified as the most effective and specific molecule, with growth inhibitory 50 (GI_50_) doses in the low micromolar range. Docking studies and biochemical assays further showed that the compound inhibited the active site of the BCR kinases Syk, Lyn, and Btk with higher efficacy than the reference kinase inhibitors [12, 13]. Here, using an extended panel of B-NHL cell lines and primary samples, we describe the full mechanism of action of this compound and report its remarkable antitumoral activity in vitro and in distinct B-NHL xenotransplant mouse models.

## Methods

### Cell lines and patients samples

Twenty-one cell lines from the different subtypes of B lymphoid neoplasm were used in this study (Table 1 and Additional file 1 Methods). All cell lines were routinely culture at 37 °C in a humidified atmosphere with 5% carbon dioxide in RPMI-1640, DMEM, or IMDM culture medium supplemented with 10–20% heat-inactivated fetal bovine serum (FBS), 2 mM glutamine, and 50 μg/ml penicillin-streptomycin (Thermo Fisher Scientific, Waltham, MA, USA). Primary tumor cells from 13 CLL patients (Additional file 1: Table S2) were used. Tumor cells were isolated, cryopreserved, and conserved within the Hematopathology collection of our institution (Hospital Clínic-IDIBAPS Biobank R121001-094), as previously described [14].

**Table 1:** Sensitivity of B lymphoid cell lines to IQS019

Cell lines	B lymphoid subtypes	*TP53* status^a^	IQS019 cytotoxic effect (referred to untreated cells)	
			1 μM, 48 h	5 μM, 48 h
DOHH-2	FL	wt	26%	100%
WSU-NHL	FL	del/mut	6%	80%
WSU-FSCCL	FL	wt	26%	77%
SC-1	FL	del/mut	4%	43%
JEKO-1	MCL	del/mut	21%	75%
MAVER-1	MCL	del/mut	15%	70%
UPN-1	MCL	del/mut	14%	66%
HBL-2	MCL	del/mut	12%	64%
MINO	MCL	del/mut	19%	64%
GRANTA-519	MCL	wt	26%	63%
Z-138	MCL	wt	18%	62%
JVM-2	MCL	wt	16%	58%
REC-1	MCL	wt	12%	48%
MEC-2	CLL	del/mut	12%	51%
JVM-13	CLL	wt	7%	46%
MEC-1	CLL	wt	9%	33%
SUDHL-16	GCB-DLBCL	del/mut	15%	47%
OCI-LY8	GCB-DLBCL	del/mut	2%	29%
SUDHL-8	GCB-DLBCL	del/mut	15%	32%
OCI-LY10	ABC-DLBCL	wt	3%	47%
U-2932	ABC-DLBCL	del/mut	15%	51%

^a^17p13 deletion was assessed by fluorescence in situ hybridization and *TP53* mutational status was analyzed by direct sequencing

*Abbreviations*: *FL* follicular lymphoma *MCL* mantle cell lymphoma, *CLL* chronic lymphocytic leukemia, *DLBCL* diffuse large B cell lymphoma

### Kinase inhibition profiling

The kinase inhibition profile of IQS019 (0.1 and 10 μM) was evaluated at Proqinase (Freiburg, Germany) using a Kinase 400-Profiler Panel, according to previously described procedures [13]. The residual activity (in %) for each compound well was calculated by using the following formula: Residual activity (%) = 100 x [(signal of compound–low control)/(high control–low control)].

### Cell-based tyrosine kinase assay

In vitro inhibitory activity of IQS019 against BCR-related kinase was determined by Advanced Cell Dynamics (San Diego, CA, USA). Briefly, the Ba/F3 murine B lymphoid cell line was transfected with either a control vector or a vector containing the kinase domain of Btk, Syk, or Lyn, rending each cell line dependent upon activity of the recombinant kinase for survival. Cells were treated for 48 h with the indicated doses of IQS019 and cell viability was monitored via ATP concentration using CellTiter-Glo assay (Promega, Madison, WI, USA). IC_50_ values were determined using the GraphPad Prism software version 5.04 (San Diego, CA, USA)

### Cell proliferation assay

Cells (4–6 x 10^5^ cells/ml) were treated for the indicated times with IQS019 or ibrutinib (Selleck Chemicals, Munich, Germany) at doses ranging from 0.1 to 20 μM, and cell proliferation was determined by a modification of the MTT (3-(4,5-dimethylthiazolyl-2)-2,5-diphenyltetrazolium bromide) reduction method.

### BCR stimulation and phospho-kinase detection

Cell lines (3–5 x 10^6^ cells) and primary CLL samples (8–10 x 10^6^ cells) were pretreated with 1 or 2.5 μM IQS019 for 90 min in FBS-free RPMI medium. Once starved, cells were incubated at 37 °C with 10 μg/ml of either anti-IgM (UPN-1, JVM-13, OCI-LY10 and primary CLL cells) or anti-IgG (DOHH-2) antibodies (Jackson Immunoresearch Laboratories, West Grove, PA, USA). Based on preliminary experiments showing a cell type-dependent variation in the optimal duration of the stimulation, cells were exposed to their respective anti-Ig for 2 min (UPN-1 and OCI-LY10 cells), 30 min (DOHH-2 and JVM-13 cells), and 15 min (CLL primary cells). Detection of phospho-Syk, phospho-lyn and phospho-Btk was carried out by western blot and flow cytometry, respectively, as detailed in Additional file 1 Methods.

### CXCL12-mediated chemotaxis

Cell lines and CLL primary cells were exposed as indicated to IQS019, with or without BCR ligation, and CXCL12-induced migration was evaluated using 24-well chemotaxis chambers containing 8 μm (cell lines) or 5 μm (primary cells) pore size inserts (Corning Life Science, Tewksbury, MA, USA), as previously described [15]. To quantify CXCR4-dependent F-actin polymerization, cells (300.000–500.000) treated as above were fixed on poly-L-lysine–coated glass coverslips with 4% paraformaldehyde, washed in PBS, permeabilized for 10 min with a solution containing 0.1% saponin (in PBS), followed by a 30 min incubation with 50 μg/ml phalloidin-TRITC (Sigma-Aldrich). Then, coverslips were washed three times with saponin 0.03%, mounted on glass slides with DAPI-containing Fluoroshield mounting medium (Sigma-Aldrich), and visualized on a Nikon H5505 microscope by means of a 60X NA oil objective (Nikon, Amsterdam, Netherlands) with the use of Isis Imaging System v5.3 software (MetaSystems GmbH, Heidelberg, Germany).

### Xenograft mouse models and immunohistochemical studies

For MCL xenotransplant model, CB17-SCID female mice (Janvier Labs, Le Genest-Saint-Isle, France) were inoculated subcutaneously with UPN-1 cells as previously described [14].Tumor-bearing mice were randomly assigned into equivalent cohorts and received a daily dose of 2 mg/kg, 10 mg/kg (i.p.), or 25 mg/kg (p.o.) IQS019-2MeSO_3_H or ibrutinib, or equal volume of vehicle, for 15 days, in a five/two (on/off) schedule. Animals were sacrificed and tumor samples were processed and stained for phospho-Histone H3 and cleaved caspase-3 as previously described [14]. Detection of phospho-Syk, phospho-Lyn and phospho-Btk was carried out from OCT tumor section as explained in Additional file 1 Methods. For systemic FL model, 12 SCID mice were intravenously inoculated via tail vein with 1.5 x 10^7^ DOHH-2 cells per mouse. One week later, animals were randomly assigned into two equivalent cohorts and treated intraperitoneally with 2 mg/kg IQS019-2MeSO_3_H or vehicle, as before. Mice were then sacrificed and immunodetection of phospho-BCR kinases was performed as detailed in Additional file 1 Methods.

### Statistical analysis

Unless otherwise specified, the data are depicted as the mean ± SD of three independent experiments. Unpaired and paired T-tests were used to obtain the statistical analysis using Graph Pad Prism software 4.0. Results were considered statistically significant when *p* < 0.05 (*, **p < 0.01, ***p < 0.001).

## Results

### Antitumor effect of the 4-aminopyrido[2,3-*d*]pyrimidine IQS019 in B lymphoid cell lines and primary samples

**Fig. 1:**
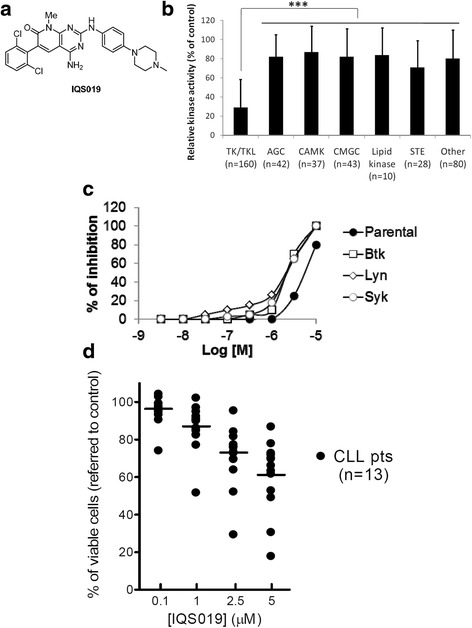
IQS019 simultaneously targets Syk, Lyn and Btk tyrosine kinases and blocks cell proliferation in distinct subtypes of B lymphoid neoplasms. **a** Chemical structure of IQS019. **b** Selectivity kinase inhibition profile of IQS019 against tyrosine kinase (TK) and tyrosine kinase-like (TKL), when compared to the other subfamilies of protein kinases (*** *p* < 0.001). **c** Effect of IQS019 on the proliferation of control (parental), Syk-, Lyn-, and Btk-overexpressing Ba/F3 cells. The different cell lines were treated with increasing dose of IQS019 and proliferation blockade was assessed by MTT assay, using untreated cells as a reference. Measured data represent the activities of duplicate determinations. **d** Effect of IQS019 on the proliferation of CLL primary cultures. Cells (2 x 10^5^) were incubated with increasing concentrations of IQS019 for 24 hours and cell viability was determined by the MTT reduction assay. Results are the mean values ± SD of four replicates, and are referred to control, untreated cells

To assess the selectivity of the kinase inhibitor IQS019 (Fig. 1a and ref [13]), we first evaluated its inhibitory property against a panel of 400 kinases, including 70 disease-relevant protein kinase mutants and 13 lipid kinases, covering about 60% of the human kinome. We found the compound to be preferentially active against tyrosine kinase (TK) and tyrosine kinase-like (TKL) families, reaching a mean residual kinase activity of 28% at a 10 μM dose, while this activity remained above 70% in all the other kinase subgroups (Fig. 1b, *** *p* < 0.001 and Additional file 1: Figure S1). Of special interest, in a set of 17 TK/TKLs, the compound was able to inhibit at least 20% of the kinase activity at the lowest dose (0.1 μM) and to achieve an almost complete kinase inactivation at the 10 μM concentration. These kinases corresponded to leucocyte-, BCR-, or T-cell receptor (TCR)-related kinases (Lyn, Blk, Lck, Src, Frk, Csk, Hck, Fyn, Btk, Syk), the member of the Tec family of non-receptor tyrosine kinases, Bmx, and other receptor tyrosine kinases with lower relevance in B-NHL, such as Ddr2, Egfr, EphA, Erbb, Fgr and Braf (Additional file 1: Table S1). Among these potential targets, a radiometric kinase activity study further showed that IQS019 had an IC_50_ in the low micromolar range for the BCR kinases Lyn (0.15 μM), Syk (1.6 μM) and Btk (2.1 μM), corresponding to those kinases able to bind the compound in their active site [13]. Accordingly, ectopic expression of each individual kinase in B lymphoid cells rendered them dependent of these kinases for their survival and increased cell sensitivity to IQS019. Indeed, while the calculated IC_50_ of the compound was 5.4 μM in parental Ba/F3 cells, this value decreased to 2.2, 1.4, and 2.2 μM in Btk-, Lyn-, or Syk-overexpressing cells, respectively (Fig. 1c). Thus, these results confirm that IQS019 is a potent tyrosine kinase inhibitor, with the unique ability to bind and to simultaneously inhibit the three BCR kinases Lyn, Syk, and Btk.

We further assessed the activity of the compound in vitro using a panel of 21 B-NHL cell lines representative of the CLL, MCL, FL and DLBCL subtypes (Table 1). We show that a 5 μM dose of the compound decreased cell proliferation in all the cell lines (range: 29–100%), being MCL and FL cells significantly more sensitive to the compound (mean cytotoxicity at 48 h: 67.2 ± 15%) than CLL cells and DLBCL cells of either activated B-cell (ABC) or germinal centre B-cell (GCB) subtype (mean cytotoxicity at 48 h: 42 ± 9%) (*p* = 0.0002). Based on these results, a set of 13 CLL primary cultures were exposed for 24 h to increasing doses of IQS019 and cell viability was measured by MTT assay. Although a high variability was observed among cases, the viability decreased in a dose-dependent manner in all the samples treated with the compound (Fig. 1d). The calculated IC_50_ was 6.1 μM in this set of samples, corresponding to the upper range of the values found in the cell lines. Similar responses were observed in FL and MCL primary cultures (data not shown). Of note, no association could be established between sensitivity to IQS019 and common cytogenetic alterations, *TP53* mutation and/or deletion, or *IGHV* mutational status (Table 1, Additional file 1: Table S2 and Figure S2a). Of interest, a 24 h treatment with a 5 μM dose of the compound induced about 35% apoptosis increase in the representative cell lines UPN-1 and DOHH-2 (Additional file 1: Figure S2b). In CLL and primary cultures (*n* = 6) the average cell death induction reached 26% (range: 9.5–51.5%), as shown in the representative cases, CLL n.2 and CLL n.10 (Additional file 1: Figure S2b and data not shown). This phenomenon was completely abrogated in the presence of the pan-caspase inhibitor Q-VD-OPh. In parallel, the analysis of phospho-histone H3 levels as a surrogate of mitotic progression indicated a notable decrease of this marker in five out of six primary CLL cases treated with the compound (Additional file 1: Figure S2c). Thus, altogether these results demonstrate that IQS019 antitumor activity in B lymphoid cells involved both a blockade in cell proliferation and the induction of a caspase-dependent cell death.

### IQS019 antagonizes constitutive and antigen-mediated BCR signaling

**Fig. 2:**
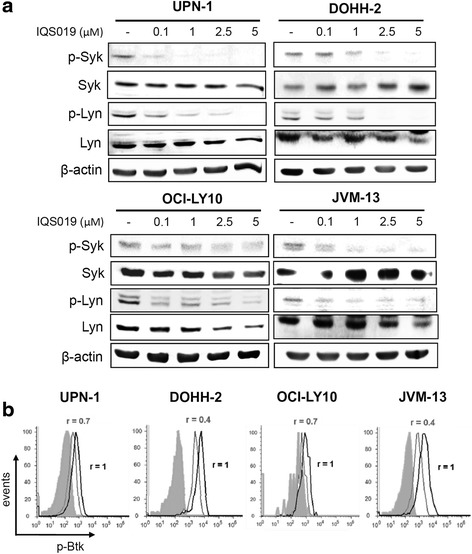
IQS019 impairs constitutive phosphorylation of Syk, Lyn and Btk tyrosine kinases in malignant B cells. **a** UPN-1, DOHH-2, OCI-LY10, and JVM-13 cells (6 x 10^6^) were treated with increasing concentrations of IQS019 for 6 h, followed by Western Blot detection of phospho(p)-Syk, and p-Lyn, using β- actin as a loading control. Shown is a representative experiment from two replicates. **b** Flow cytometry analysis of p-Btk levels in cell lines treated as before. A PE-labeled mouse IgG1 κ was used as an isotype control (*grey* filled histogram). Indicated are the relative median fluorescence intensity (*r*) values observed after treatment with 5 μM IQS019 (*grey curves*), and referred to control untreated cells (*black curves*)

Based on the above results, we analyzed the effect of IQS019 on the phosphorylation status of Syk, Lyn and Btk in four cell lines representative of MCL (UPN-1), FL (DOHH-2), CLL (JVM-13), and DLBCL (OCI-LY10) subtypes. Cells were incubated for 6 h with increasing concentrations of IQS019 and phosphorylation levels of Syk and Lyn at their respective Tyr352 and Tyr396 residues, were evaluated by Western blot, while Btk phosphorylation at Tyr223 residue was analyzed by flow cytometry. As observed in Fig. 2a, IQS019 treatment led to a dose-dependent dephosphorylation of Syk and Lyn in the four cell lines tested. Consistent with the cytotoxicity of the compound (Table 1), a complete dephosphorylation of the two kinases was observed in UPN-1 and DOHH-2, while a slight, persistent phosphorylation of both Syk and Lyn was detected in OCI-LY10 and JVM-13 cells (Fig. 2a). Regarding Btk phosphorylation, flow cytometry analysis showed a 30% (UPN-1 and OCI-LY10) and a 60% (DOHH-2 and JVM-13) decrease in the relative mean fluorescence intensity ratio (r) of phospho-Btk signal in cells exposed to a 5 μM dose of the compound (Fig. 2b).

**Fig. 3:**
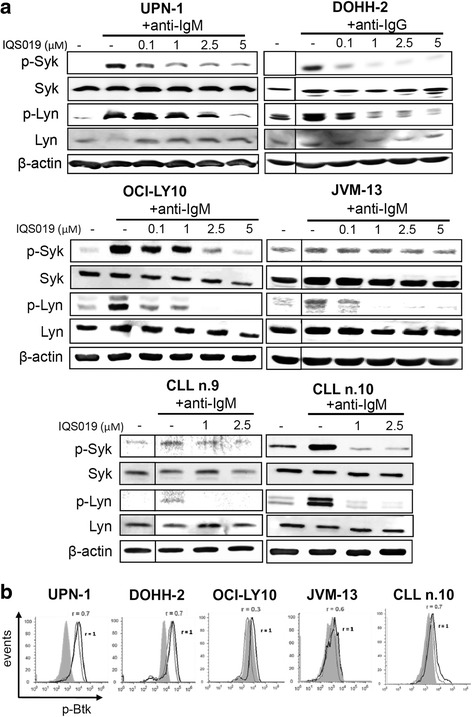
IQS019 overcomes anti-Ig-mediated BCR activation in B lymphoid cell lines and primary samples. Representative cell lines and primary cultures were pre-treated for 1.5 h with increasing concentrations of IQS019, followed by anti-Ig-mediated BCR ligation, as described in methods section. **a** Expression levels of p-Syk and p-Lyn were analyzed by Western Blot, as previously, using β-actin as a loading control. Shown is a representative experiment from two replicates. **b** p-Btk levels were analyzed as previously by flow cytometry in B lymphoid cell lines and primary cultures, pre-stimulated with their respective anti-Ig and either untreated (*black curves*) or exposed to 5 μM IQS019 (*grey* *curves* ). Shown is a representative experiment from a CLL patient out of a series of six cases

In a second step, the four previous cell lines and two representative primary CLL cases were BCR-stimulated with their corresponding anti-Ig in the presence of increasing concentrations of IQS019, and phospho-Syk, phospho-Lyn and phospho-Btk levels were analyzed as above. As shown in Fig. 3a, BCR ligation induced an increase in the phosphorylation levels of Syk and Lyn in all the samples tested, that was hampered by IQS019 in a dose-dependent manner. Remarkably, a dose of IQS019 as low as 1 μM was sufficient to completely counteract the anti-IgM-mediated activation of Syk and Lyn in the highly IgM-responsive (unmutated *IGHV*) CLL sample showing the greatest efficacy of the stimulation (CLL#10, Fig. 3a). Similarly, IQS019 efficiently counteracted Ig-induced Btk phosphorylation in cell lines, as shown by a 30 to 70% reduction in relative phospho-Btk levels (Fig. 3b). In CLL primary cells, for all but 1 cases out of the 6 examined, IQS019 achieved a 30% reduction in phospho-Btk levels (*p* = 0.0005), as shown in the representative CLL no.10 (Fig. 3b and data not shown). Of note, in the representative cell line UPN-1, Syk-dependent phosphorylation of Btk at Tyr551 was negligible upon BCR triggering and remained unaffected in the presence of IQS019 (data not shown), suggesting that IQS019-mediated inhibition of Btk requires a direct interaction of the compound with the kinase, rather than an indirect, Syk-mediated signal transduction. Altogether, these results suggest than IQS019 counteracts both constitutive and antigen-induced BCR signaling in B lymphoid cell lines and primary cells.

### IQS019 inhibits CXCL12-mediated migration of malignant B cells

**Fig. 4:**
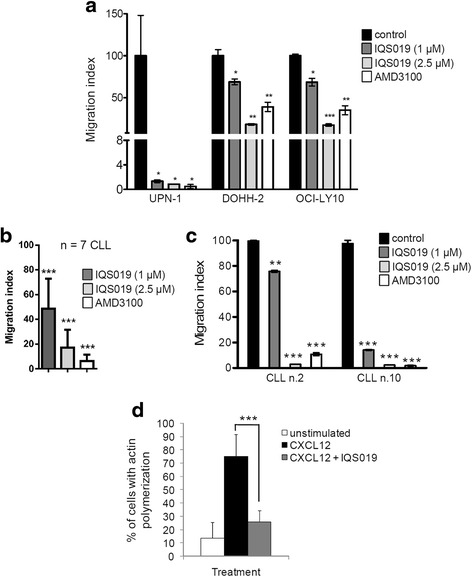
IQS019 interferes with malignant B cell chemotaxis. Representative, CXCR4-expressing B lymphoid cell lines (**a**) and a set of *n* = 7 CLL primary samples (**b**) were exposed for 1.5 h to 1 or 2.5 μM IQS019, with or without Ig-mediated BCR stimulation, followed by cytofluorimetric recounting of cells migrated towards recombinant CXCL12 in a 4-h transwell assay. Treatment with the CXCR4 antagonist AMD3100 (40 μM) was used as a control of chemotaxis blockade. Expressed are the ratios between CXCL12-dependent and CXCL12-unspecific migration. **c** Cell migration profiles from two representative CLL cases are shown. **d** A set of six CLL cultures were treated as above with CXCL12 +/- IQS019 and stained with phalloidin-TRITC. Cells with high levels of polymerized F-actin were recounted for each condition by two independent reviewers. Shown are the mean results obtained from the six cases. Statistical significance: * *p* < 0.05, ** *p* < 0.01, *** *p* < 0.001

Migration of neoplastic B cells has been shown to be heavily affected upon exposure to drugs targeting the BCR-associated kinases, as these latest tightly regulate the re-organization of the cytoskeleton required for cell chemotaxis [15]. Thus, we evaluated the effect of IQS019 on the migratory capacity of malignant B cells, using a CXCL12-dependent chemotaxis assay with 3 cell lines harboring detectable levels of CXCR4 (Additional file 1: Figure S3) and in a set of seven CLL primary samples, either untreated or pre-treated with IQS019 or with the standard CXCR4 antagonist AMD3100. The migration induced by recombinant CXCL12 in MCL, FL and DLBCL cell lines was significantly inhibited by the compound at all the doses tested (Fig. 4a). The statistical significance of this effect was higher at the 2.5 μM than at the 1 μM dose in DOHH-2 and OCI-LY10 cells, while an almost complete inhibition of cell migration was achieved in UPN-1 cells at the lowest dose. In the case of CLL primary cells, since the stimulation of BCR has been shown to facilitate CXCL12-mediated migration [15], we evaluated the activity of IQS019 after BCR crosslinking. As shown in Fig. 4b, c, IQS019 significantly overcame IgM-activated, CXCL12-dependent chemotaxis in all the primary samples tested, either at the 1 μM dose (mean inhibition: 51.5%; range: 23.9–85.5%; *p* = 0.0013) or at the 2.5 μM dose (mean inhibition: 82.9%; range: 63.4–97.6%; *p* < 0.0001), when compared to untreated control cells. Accordingly, the mean fraction of cells with detectable F-actin polymerization shifted from 13.8% in control cells to 75.1% after CXCL12 stimulation, and was lowered down to 25.9% in the presence of IQS019 (Fig. 4d, *** *p* = 0.0003). Of special interest, when comparing with AMD3100, IQS019 showed similar, or even superior anti-migratory activity (Fig. 4a, b and c). These results indicate that IQS019-mediated inhibition of BCR upstream kinases may interfere with B cell chemotaxis and tumor cell dissemination.

### IQS019 is safe and impairs tumor outgrowth and malignant B cell homing to spleen in vivo

**Fig. 5:**
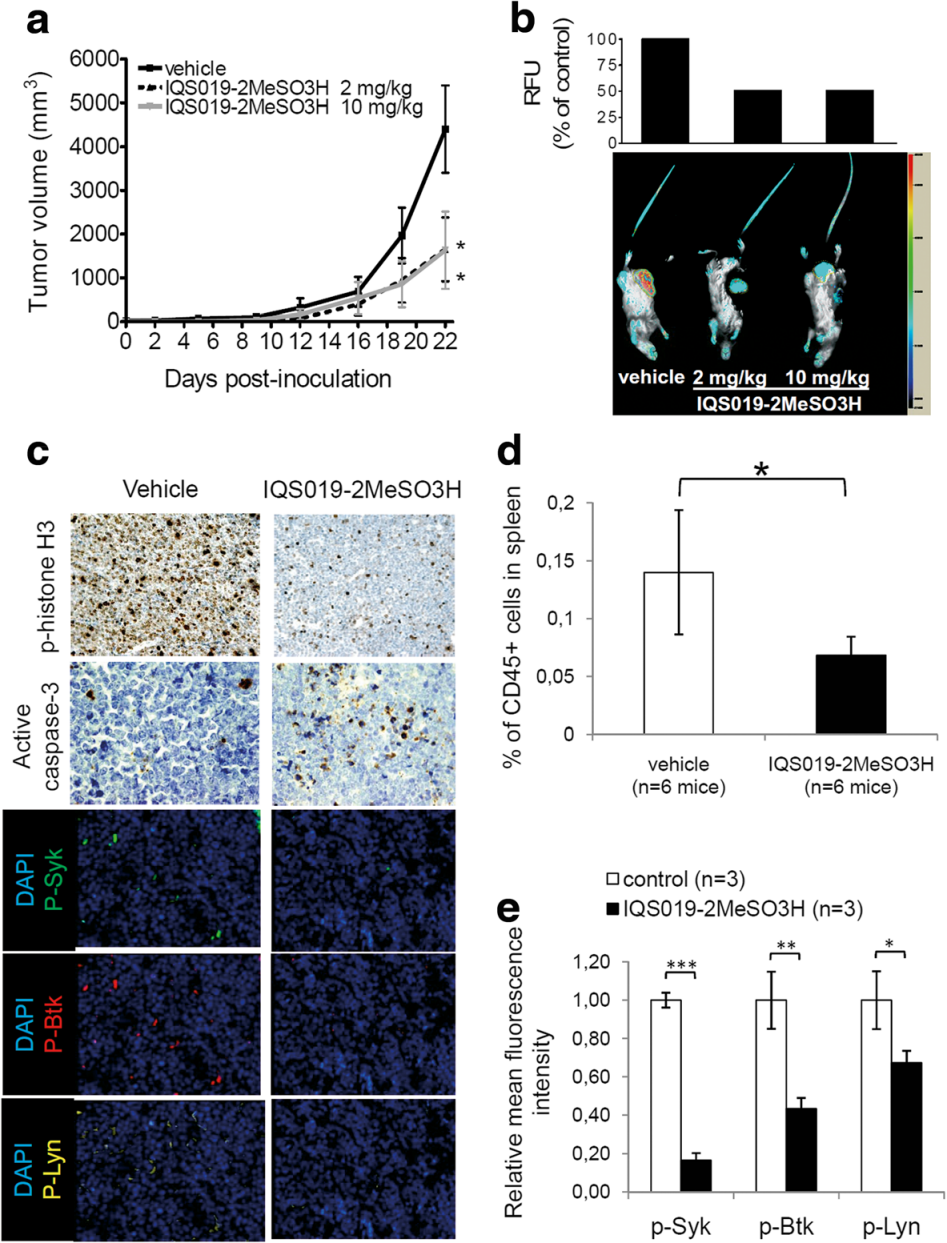
IQS019 impairs tumor growth and homing of B lymphoid cells to spleen *in vivo*. **a** SCID mice were inoculated with UPN-1 cells and began treatment at day 10 post-inoculation with 2 or 10 mg/kg IQS019-2MeSO_3_H or equal volume of vehicle. IQS019-2MeSO_3_H was administrated 5 days a week for 2 weeks. **b** *Lower panel*, intratumoral glucose uptake was evaluated in representative mice injected intravenously with an IR800-labeled 2-deoxy glucose probe 24 h prior sacrifice, and visualized with an Odyssey infra-red scanner (Li-Cor). *Upper panel*, relative fluorescence quantification by means of the Image Studio software shows markedly reduced glucose uptake in tumor masses from mice receiving IQS019-2MeSO_3_H, when compared to vehicle-treated animals. **c** Immunostaining of consecutive sections from representative UPN-1-derived tumors, showing the decrease of proliferation and the induction of apoptosis accompanying the downregulation of p-Syk, p-Lyn and p-Btk upon IQS019-2MeSO_3_H treatment (magnification 200X). **d** Mice were injected intravenously with DOHH-2 cells and, after one week, received a 2 mg/kg dose of IQS019-2MeSO_3_H or vehicle, daily, for up to 14 days. Mice were then sacrificed and human (CD45+) malignant B cells were recounted from spleen as described in methods section (statistical significance: * *p* < 0.05). **e** In each treatment group, CD45+/CD20+ human B cells isolated from *n* = 3 representative animals, were labeled with anti-p-Syk, anti-p-Lyn, or anti-p-Btk antibody, and fluorescence was recorded on a cytometer. Shown are the relative *r* values among control and vehicle group, calculated for each phospho-kinase

In order to validate the activity of IQS019 in vivo, we first synthesized the salt form of the compound, thereafter labeled as IQS019-2MeSO3H, and evaluated its single-dose toxicity over 14 days after intravenous administration in healthy immunodeficient (SCID) mice (details in Additional file 1 Methods). As the maximum tolerated dose was not reached, 2 and 10 mg/kg doses were selected for further in vivo experiments. We then developed two different, complementary xenotransplant animal models of the two entities showing increased sensitivity to the compound in vitro, i.e., MCL and FL. Heterotopic MCL tumors were generated in SCID mice subcutaneously inoculated with UPN-1 cells, while a systemic (i.e., characterized by homing of tumor B cells from peripheral blood to spleen) FL tumor model was obtained by intravenous injection of DOHH-2 cells in SCID mice. As shown in Fig. 5a, after two weeks of treatment, mice bearing MCL tumors and dosed with IQS019-2MeSO_3_H showed a 63% reduction in tumor volume, when compared to the vehicle group (*p < 0.05). There was not subsequent improvement of the anti-tumor activity of the compound between the 2 mg/kg and the 10 mg/kg dosing, suggesting that optimal activity was reached at the lowest dose. Consistently, tumor metabolism was similarly decreased in both treatment groups, as glucose uptake fell to 50–52% in tumors from all IQS019-2MeSO_3_H-exposed animals, irrespective of the dose (Fig. 5b). This effect was closely related to the inhibition of the three BCR-related kinases Syk, Lyn, and Btk, as shown by a complete reduction of their phosphorylated forms in the drug-treated specimens, when compared to the control group (Fig. 5c). Immunohistochemical analysis of representative tumor sections further revealed that IQS019 therapy efficiently reduced the mitotic index and induced apoptosis in UPN-1-derived tumors, as shown by a decreased labeling of phospho-histone H3 and an intracellular increase in the activated form of caspase-3 (Fig. 5c).

In the systemic DOHH-2 mouse model, mice dosing was initiated at day 7 post-inoculation, with a 2 mg/kg IQS019-2MeSO_3_H regimen, daily, for 15 days. Once inoculated, FL cells rapidly migrate to the spleen [16]. Therefore, at the end of the procedure, entire spleens were processed, and the presence of malignant B cells was evaluated by labeling with anti-human CD45 antibody and tumor cell recounting on a flow cytometer. IQS019-2MeSO_3_H treatment induced a 52% reduction in tumor cell infiltration into the spleen, when compared to vehicle group (Fig. 5d, * *p* = 0.01). Accordingly, the r fluorescence values of phospho-Syk, phospho-Btk, and phospho-Lyn, decreased by 83, 57, and 33% in tumors B cells purified from IQS019-treated animals (Fig. 5e). Altogether, these results demonstrate that IQS019 is safe and exhibits in vivo efficacy against MCL and FL tumor burden, involving the inhibition of BCR signaling and the blockade of tumor cell homing to lymphoid compartment.

### IQS019 shows superior anti-tumor activity than ibrutinib in vitro and in vivo

**Fig. 6:**
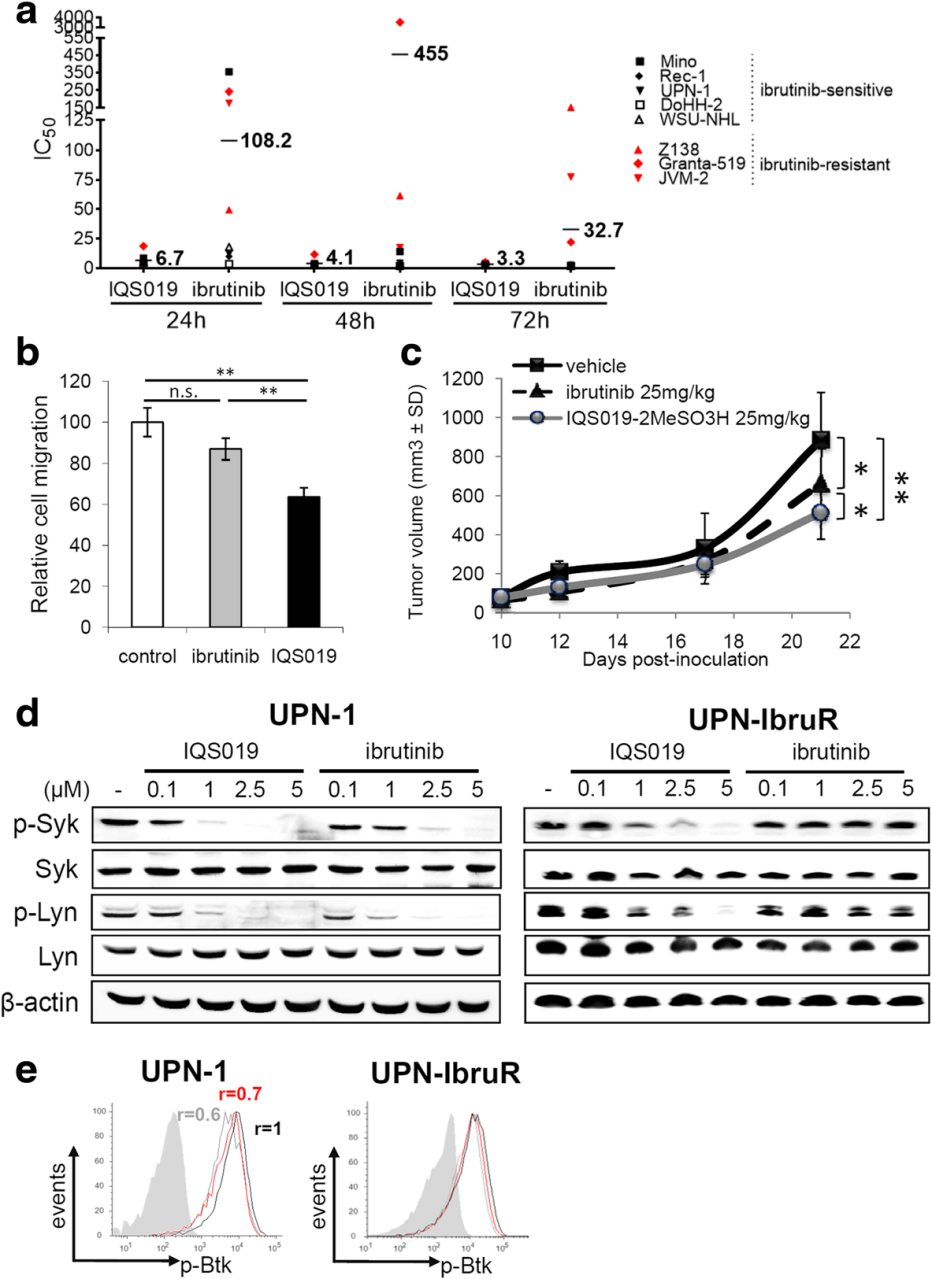
Improved in vitro and in vivo activity of IQS019 *vs* the Btk inhibitor ibrutinib. **a** A panel of eight B-NHL cell lines was incubated with increasing concentrations of IQS019 or ibrutinib for 24, 48, and 72 h and IC_50_ was calculated as previously. **b** IQS019 has improved anti-migratory activity. UPN-1 cells were exposed to 5 μM IQS019 or ibrutinib for 90 min followed by evaluation of CXCL12-dependent cell chemotaxis, as described in methods section. Statistical significance: ** *p* < 0.01. **c** Orally administered IQS019 impairs tumor outgrowth more efficiently than ibrutinib. SCID mice were inoculated with UPN-1 cells subcutaneously and, at day 10 post-inoculation, started to be dosed p.o., 5 days a week, for two weeks, with 25 mg/kg IQS019-2MeSO3H, 25 mg/kg ibrutinib or equal volume of vehicle (*n* = 10 animals per group). Tumor volumes were recorded as above. Statistical significance: * *p* < 0.05, ** *p* < 0.01. **d** Western blot analysis of phospho-Lyk, phospho-Syk and (**e**) cytofluorimetric analysis of phospho-Btk in UPN-1 and UPN-IbruR cells treated for 6 h with indicated doses of IQS019 or ibrutinib. For phospho-Btk analysis, cells were exposed to a single dose of each agent (5 μM) 90 min prior to IgM stimulation and cell labeling. Indicated are the *r* values observed after treatment with IQS019 (*grey curves*) or ibrutinib (*red curves*), and referred to control IgM-stimulated cells (*black curves*). Isotype controls are represented by *grey* filled histograms. Shown are representative experiments from two replicates

We previously reported that IQS019 presented an increased anti-proliferative activity in vitro when compared to ibrutinib, in a single MCL cell line and at a single time point [13]. To confirm this preliminary experiment, we compared by MTT assay the anti-proliferative effect of IQS019 and ibrutinib at 24, 48, and 72 h, using doses ranging from 0.5 to 10 μM, in a panel of eight cell lines that included the ibrutinib-sensitive MINO, REC-1, UPN-1, DOHH-2, and WSU-NHL and the ibrutinib-resistant Z-138, GRANTA-519, and JVM-2 cells. Figure 6a shows that the mean IC_50_ of ibrutinib remained significantly high (i.e., > 10 μM) in this set of cell lines, even after a 72-h drug exposure, mainly due to the high values observed in the resistant cell lines (152.4 μM for Z-138, 22.1 μM for GRANTA-519 and 77.4 μM for JVM-2). In contrast, IQS09-mediated proliferation blockade was almost completely reached in all the cell lines after only 24 hours, with a mean IC_50_ of 6.7 μM (range: 2.7–8.7 μM), which decreased down to 4.1 μM (range: 2.2–11.7 μM) and 3.3 μM (range: 2.2-5.1 μM) at 48 h and 72 h, respectively. Of particular interest, after 72 h these IC_50_ values were much lower in Z-138 (4.5 μM), GRANTA-519 (5.1 μM), and JVM-2 cells (4.1 μM) than observed after ibrutinib treatment. Accordingly, while a short exposure to ibrutinib only marginally affected CXCL12-dependent cell migration in the UPN-1 cell line, this process was blocked up to 39% in cells cultured with IQS019 (Fig. 6b). To validate these observations in in vivo settings, MCL tumor-bearing mice were treated with a standard 25 mg/kg dose of ibrutinib [17], the equivalent dose of IQS019-2MeSO_3_H, or vehicle. While ibrutinib allowed to a significant 25.1% reduction in tumor growth after 2 weeks of treatment (* *p* = 0.049), IQS019-2MeSO_3_H showed superior activity, as it could inhibit the tumor outgrowth up to 42% when compared to vehicle group (Fig. 6c, ** *p* = 0.006, * *p* = 0.048). At the pharmacokinetic level, while both compounds presented similar half-life and C_max_ values in mice after a single oral administration, the total drug exposure over time was considerably improved in the case of IQS019-2MeSO_3_H, as shown by a 12 fold increase in the AUC value. Consequently, the global bioavailability dropped from 2 to 4% in the case of ibrutinib, to about 70% in the case of IQS019-2MeSO_3_H (Additional file 1: Figure S4b and Table S3), thus suggesting that a better PK profile may account for the improved activity of IQS019 *vs* ibrutinib in vivo.

To unravel at the molecular level the mechanisms underlying this superior activity of IQS019 over the Btk inhibitor, we established an ibrutinib-resistant cell line designated UPN-IbruR, derived from the parental UPN-1 by repeated drug selection (Additional file 1 Methods). When compared with the parental cell line, UPN-IbruR presented approximately a 10-fold increase in the ibrutinib IC_50_ after 72 h of treatment (24.6 vs 2.4 μM for parental cells), with negligible difference in IQS019 IC_50_ (5.6 vs 2.3 μM for parental cells) (Additional file 1: Figure S5a).The ibrutinib resistance phenotype of UPN-IbruR cells was not associated to mutations in *BTK* or *PLCG2* genes, which both harbored a wild type sequence (Additional file 1: Figure S5b), but may rather be associated to the activation of non-canonical NF-κB pathway, as suggested by the overexpression of p52 (Additional file 1: Figure S5c). While a similar dose-dependent decrease in phospho-Lyn and phospho-Btk levels was found in IQS019- and in ibrutinib-treated UPN-1 cells, the expression of phospho-Syk was almost completely lost only in cells exposed to 1 μM IQS019 (Fig. 6d,e). In sharp contrast, in UPN-IbruR cells, the Btk inhibitor failed to modulate the phosphorylation of the three kinases, while IQS019 showed significant inhibitory activity of phospho-Syk and phospho-Lyn at a dose as low as 1 μM (Fig. 6d). However, the compound was unable to downregulate phospho-Btk (Fig. 6e), suggesting that in ibrutinib-resistant cells, the capacity of IQS019 to inhibit Syk and Lyn may allow the compound to maintain a significant antitumoral activity independent of the expression of a non-druggable form of Btk. Altogether, these results point out a significant superior antitumoral activity of pleiotropic BCR kinase targeting by IQS019 over the sole inhibition of Btk, in in vitro and in vivo models of B-NHL.

## Discussion

BCR has recently emerged as a central oncogenic pathway that promotes growth and survival in various lymphoma subtypes [8]. Constitutive activation of the three BCR-related kinases Syk, Lyn, and Btk have been well documented in CLL [18–20], MCL [21, 22], and FL [23] cells, while chronic BCR signaling has been reported in the ABC subtype of DLBCL [5]. Consistently, BCR kinase inhibitors constitute promising therapeutic strategies in these different entities. Among these novel agents, the first-in-class Btk inhibitor ibrutinib has achieved high response rates (43–71%) in relapsed/refractory CLL, MCL and ABC-DLBCL patients, while its activity was less pronounced in FL patients (37% overall response rate) [24–27]. A small fraction of patients develop progressive disease after initial response to this agent [25, 27], in relation with the acquisition of mutations at the ibrutinib binding site (C481S) of Btk, or in the *PLCγ2* gene [9–11]. Resistance to ibrutinib may also involve a lower dependency of malignant B cells toward Btk itself, than other downstream components of the pathway, like the Syk/Lyn-dependent kinase Erk [28]. Accordingly, the Syk inhibitor fostamatinib and the Src inhibitor dasatinib have also shown efficacy in relapsed/refractory B-NHL [29, 30].

Following these observations, and in an effort to improve the therapeutic modulation of BCR signaling, we previously screened a library of compounds derived from pyrido[2,3-*d*]pyrimidines, for their capacity to bind to the active sites of Btk, Syk and/or Lyn [31]. We identified IQS019 (compound 19) as a unique molecule with affinity for the three BCR kinases [13]. In the present work, we confirm the inhibitory property of the compound against Btk, Syk and Lyn, as well as its selective antitumoral effect in B lymphoid cells, especially in MCL and FL cell lines, and independently of the response to ibrutinib. Our results suggest that IQS019 can counteract both chronic and tonic BCR signaling, as it shows similar antiproliferative activity in DLBCL cell lines from both the GCB and ABC subtype, which are respectively dependent for their survival on tonic (Syk/PI3K-mediated) and chronic (Syk/Btk-mediated) BCR signaling [8, 32–34]. This property might confer to IQS019 a greater activity than ibrutinib, which is preferentially active against tumors that rely on chronic active BCR signaling [8]. Beside Btk, the direct inhibitory activity of IQS019 towards Syk and/or Lyn phosphorylation may also explain the capacity of the compound to activate apoptosis in vitro and in vivo, as pharmacological inhibition of Syk, has been reported to elicit the apoptotic cascade in preclinical models of DLBCL and CLL [35, 36]. Also, probably thanks to its apoptogenic property and specificity, IQS019 salt is found to be significantly active and safe at a dose of 2 mg/kg/day, which is much lower than the reported active concentrations of fostamatinib, dasatinib or ibrutinib in mouse models of lymphoid neoplasms [37–39], thus predicting a probable low incidence of secondary adverse effects of the compound.

Another downstream event regulated by Btk, Syk, and Lyn is the chemokine-mediated B cell migration, a process essential to tumor B cell survival [40]. We show that IQS019 is able to impair in vitro cell migration towards CXCL12 in cell lines and primary samples, in both basal and anti-Ig-stimulated cultures. This property may be responsible, at least in part, for the reduced infiltration of tumor cell observed in FL-bearing mice dosed with the compound. Beside this effect, IQS019-mediated inhibition of Syk, Lyn, and Btk may further impair tumor maintenance and B cell homeostasis in vivo, which are largely dependent on the coordinated activity of the three kinases [41].

## Conclusions

In summary, we describe IQS019 as a new and unique BCR kinase inhibitor able to counteract both constitutive and ligand-dependent activation of the BCR pathway in in vitro and in vivo models of B lymphoid neoplasms. Thanks to the unique capacity of the compound to inhibit the three upstream BCR kinases Lyn, Syk, and Btk, this study may offer a glimpse into possible application for the treatment of the most prevalent subtypes of B-NHL, including those low responders to current BCR kinase inhibitors.

## Additional file

Additional file 1:
**Figure S1**. IQS019 tyrosine kinase inhibitory profiling. Tyrosine kinase (TK) and tyrosine kinase-like (TKL) kinome tree was elaborated on the basis of residual in vitro kinase activity upon exposure to 100 nM or 1 μM IQS019, by means of Kinome Render software (http://bcb.med.usherbrooke.ca/kinomerender.php). **Figure S2**. Sensitivity of CLL primary cases to IQS019 is independent of IGHV mutational status and involves a caspase-dependent cell death process. (a) CLL primary cells, 9 of them with ummutated (UM) and 6 with mutated (M) IGHV gene, were treated with increasing concentrations of IQS019 for 24h. Cell viability was determined by MTT method. Shown are the median values from each CLL group (UM and M), referred to control, untreated cells. (b) IQS019 induces caspase-dependent cell death in MCL (UPN-1) and in FL (DOHH-2) cell lines, as well as in two representative CLL primary cultures. Cells were exposed for 24 hours to 5 μM IQS019, in the presence of absence of the pan-caspase inhibitor Q-VD-OPh (10 μM). Apoptosis was determined by simultaneous cytofluorimetric detection of Annexin-V and caspase-3/7 activity. (c) A set of 6 CLL primary cultures were treated with IQS019 as indicated, followed by Western Blot detection of phospho-histone H3 (p-H3), using β- actin as a loading control. **Figure S3**. Flow cytometry determination of CXCR4 membrane expression in B-NHL cell lines. Four representative cell lines were stained with a PE-labeled anti-CXCR4 antibody and analyzed on an Attune cytometer. CXCR4-specific signal (black curves) and isotypic control (grey filled curve) are represented. **Figure S4**. Safety and PK properties of IQS019-2MeSO3H in mice. (a) Twenty SCID mice (10 males and 10 females) received a single intravenous injection of IQS019-2MeSO3H at a 2 mg/kg, 10 mg/kg, or 50 mg/kg dose, or equivalent volume of vehicle, and animal weight was recorded at days 1, 3, 4, 7, 11, 14, 18 and 21 post-treatment. (b) Mean plasma concentration of IQS019-2MeSO3H in ICR mice over the time, after a single p.o. administration of a 25 mg/kg dose of the compound. **Figure S5**. Comparison of parental and ibrutinib-resistant derived B-NHL cell line. (a) Dose-response of the UPN-1 parental, and UPN-IbruR derived cell line exposed for 72 hours to increasing concentrations of ibrutinib or IQS019. (b) BTK and PLCG2 exon sequencing in UPN-IbruR cells. (c) Western blot detection of the alternative NF-κB pathway component, p52, in UPN-1 and UPN-IbruR cells. β-actin was used as a loading control. (DOC 3279 kb)

## Abbreviations

ABC: Activated B-cell
AGC: A, G, and C protein kinase group
BCR: B cell receptor
B-NHL: B-cell non-Hodgkin lymphoma
Btk: Bruton’s kinase
CAMK: Ca2+/calmodulin-dependent protein kinase
CLL: Chronic lymphocytic leukemia
CMGC: Cyclin-dependent (CDKs), mitogen-activated, glycogen synthase and CDK-like protein kinase group
DLBCL: Diffuse large B-cell lymphoma
FBS: Fetal bovine serum
FL: Follicular lymphoma
GCB: Germinal centre B-cell
GI_50_: Growth inhibitory 50
Lyn: Lck/Yes novel tyrosine kinase
MCL: Mantle cell lymphoma
PLCγ2: Phospholipase Cγ2
STE: Mitogen-activated protein kinase cascade component
Syk: Spleen tyrosine kinase
TK: Tyrosine kinase
TKL: Tyrosine kinase-like
