# Supplementary material for: Activity of the novel BCR kinase inhibitor IQS019 in preclinical models of B-cell non-Hodgkin lymphoma
Source: J Hematol Oncol. 2017 Mar 31;10:80. doi: 10.1186/s13045-017-0447-6 (PMC5374673; doi:10.1186/s13045-017-0447-6)
Supplement: Additional file 1: — Figure S1. IQS019 tyrosine kinase inhibitory profiling. Tyrosine kinase (TK) and tyrosine kinase-like (TKL) kinome tree was elaborated on the basis of residual in vitro kinase activity upon exposure to 100 nM or 1 μM IQS019, by means of Kinome Render software (http://bcb.med.usherbrooke.ca/kinomerender.php). Figure S2. Sensitivity of CLL primary cases to IQS019 is independent of IGHV mutational status and involves a caspase-dependent cell death process. (a) CLL primary cells, 9 of them with ummutated (UM) and 6 with mutated (M) IGHV gene, were treated with increasing concentrations of IQS019 for 24h. Cell viability was determined by MTT method. Shown are the median values from each CLL group (UM and M), referred to control, untreated cells. (b) IQS019 induces caspase-dependent cell death in MCL (UPN-1) and in FL (DOHH-2) cell lines, as well as in two representative CLL primary cultures. Cells were exposed for 24 hours to 5 μM IQS019, in the presence of absence of the pan-caspase inhibitor Q-VD-OPh (10 μM). Apoptosis was determined by simultaneous cytofluorimetric detection of Annexin-V and caspase-3/7 activity. (c) A set of 6 CLL primary cultures were treated with IQS019 as indicated, followed by Western Blot detection of phospho-histone H3 (p-H3), using β- actin as a loading control. Figure S3. Flow cytometry determination of CXCR4 membrane expression in B-NHL cell lines. Four representative cell lines were stained with a PE-labeled anti-CXCR4 antibody and analyzed on an Attune cytometer. CXCR4-specific signal (black curves) and isotypic control (grey filled curve) are represented. Figure S4. Safety and PK properties of IQS019-2MeSO3H in mice. (a) Twenty SCID mice (10 males and 10 females) received a single intravenous injection of IQS019-2MeSO3H at a 2 mg/kg, 10 mg/kg, or 50 mg/kg dose, or equivalent volume of vehicle, and animal weight was recorded at days 1, 3, 4, 7, 11, 14, 18 and 21 post-treatment. (b) Mean plasma concentration of IQS019-2MeSO3H in ICR mice ov [file 13045_2017_447_MOESM1_ESM.doc]

# Additional file 1

# SUPPLEMENTAL METHODS

**Cell line authentication**

JEKO-1, MINO, JVM-2, REC-1 and JVM-13 cell lines were obtained from ATCC cell bank (LGC Standards, Teddington, UK). DOHH-2, WSU-NHL, WSU-FSCLL, SC-1, GRANTA-519, MEC-2, MEC-1, SUDHL-16, SUDHL-8 and U-2932 cell lines were purchased at DSMZ (Braunschweig, Germany). MAVER-1, UPN-1, HBL-2, Z-138, OCI-LY8 and OCI-LY10 were provided, respectively, by Dr A. Zamo (University of Verona, Verona, Italy), Dr A. Turhan (Institut Gustave Roussy, Villejuif, France), Dr M. Dreyling (University Hospital, Munich, Germany), Dr E. Ortega-Paino (Lund University, Lund, Sweden), Dr M. Raffeld (National Cancer Institute Bethesda, MD, USA) and Dr A. Staiger (Dr. Margarete Fischer-Bosch Institute of Clinical Pharmacology, Stuttgart, Germany). Cell line authentication was performed upon reception by short tandem repeat (STR) profiling, using AmpFlSTR identifier kit (Thermo Fisher), and based on available STR profiles. This analysis was then repeated every 6 months and up to 4 months prior to the submission of the present manuscript. Mycoplasm infection was routinely tested by PCR.

**Apoptosis assay**

# Cells (4-6 x 10^5^ cells/ml) were treated with IQS019 and, when specified, were preincubated for 1 hour with 10 μM of the pan-caspase inhibitor Q-VD-OPh (Merck). Apoptosis was determined by dual labeling of phosphatidylserine exposure and caspase activity by means of Annexin-V Pacific Blue and CellEvent caspase-3/7 Green (Thermo Fisher), respectively, followed by the analysis of 10.000 events on an Attune acoustic focusing cytometer.

# Preparation of IQS019 soluble salt and toxicity assay in SCID mice. IQS019-2MeSO_3_H was synthesized according to the following procedure: 382.8 mg (0.75 mmol) of IQS019 were dissolved in 50 ml of acetone and then 144.3 mg (1.5 mmol) of methanesulfonic acid were added. The resulting solution was stirred at room temperature for 2 hours and then cold diethyl ether was added and the resulting precipitated was filtered, washed with cold diethyl ether and dried *in vacuo* over phosphorus pentoxide to afford 456.7 mg (0.65 mmol, 87%) of 4-amino-6-(2,6-dichlorophenyl)-8-methyl-2-(4-(4-methylpiperazin-1-yl)phenylamino)pyrido[2,3-d]pyrimidin-7(8H)-one dimesylate (IQS019-2MeSO_3_H)as a yellowish solid. ^1^H NMR (400 MHz, DMSO-*d_6_*) δ 9.53 (br s, 2H), 9.34 (br s, 1H), 8.09 (s, 1H), 7.70 (d, *J* = 9.1 Hz, 2H), 7.59 – 7.56 (m, 2H), 7.44 (dd, *J* = 8.7, 7.5 Hz, 1H), 7.43 (br s, 2H), 6.98 (d, *J* = 9.2 Hz, 2H), 3.77 (d, *J* = 13.5 Hz, 2H) 3.59 (s, 3H), 3.52 (d, *J* = 11.9 Hz, 2H) 3.24 – 3.11 (m, 2H), 2.98 – 2.84 (m, 5H), 2.34 (s, 6H).

# Four groups consisting of 4 animals (2 males and 2 females per group), each received a 2 mg/kg, a 10 mg/kg, or a 50 mg/kg dose of the compound, or equal volume of vehicle (saline solution) were evaluated. Viability/mortality, motility, hair appearance and body weights of the animals were recorded during the first 30 min and at approximately 3 and 5 h after administration on test day 1, and once daily between days 2 and day 14. At the end of the observation period, no mortality or alteration of vital parameters, including body weight was recorded in animals administered with either the saline solution or the compound (supplemental Fig. S4a). Macroscopic examination of the animals after sacrifice revealed no alteration of the principal organ systems.

***In vitro* evaluation of BCR-related kinase phosphorylation**

For the detection of phospho-Btk in cell lines, 0.5 x 10^6^ cells were fixed with 4% PFA for 15 min on ice, washed once, and permeabilized with pure methanol for 10 min at -20ºC. In the case of CLL primary cells, phosphatase-mediated dephosphorylation of Btk was prevented by adding 3.3 mM hydrogen peroxide during the last 2 min of IgM stimulation, followed by a wash in cold PBS before cell fixation with PFA as before and cell permeabilization with ethanol 70% for 2 hours. Cells were then washed twice and stained for 15 min at RT with a phycoerythrin (PE)-labeled anti-Btk-phosphoTyr223 antibody (clone N35-86) or a mouse IgG1 κ isotypic control (Becton Dickinson), in PBS + 0.5% BSA, followed by analysis on an Attune acoustic focusing cytometer (Thermo Fisher).

# Phospho-Syk and phospho-Lyn protein levels were analyzed by SDS-PAGE, using whole cell extracts obtained by lysing 3-5 x 10^6^ cells on ice for 30 min in Triton buffer (20 mM Tris-HCl ph 7.6, 150 Mm NaCl, 1 mM EDTA, 1% Triton X-100) supplemented with protease and phosphatase inhibitors. Membranes were incubated with anti-Syk-phosphoTyr352 (Cell Signaling Technology, Danvers, MA, USA), anti-Lyn-phosphoTyr396 (Abcam, Cambridge, UK), and anti-β-actin (Sigma-Aldrich, Saint-Louis, MO, USA) primary antibodies, followed by appropriate anti-rabbit secondary antibody (Cell Signaling Technology). Chemiluminiscence detection was done using the ECL system (Thermo Fisher) and visualized on a mini-LAS4000 device using Image Gauge software (Fujifilm, Valhalla, NY, USA). In order to avoid signal saturation consequent to BCR ligation, exposure time of membranes containing protein extracts from anti-Ig- treated UPN-1, DOHH-2 and OCI-LY10 cells, were substantially reduced.

# Detection of phospho-Syk, phospho-Lyn and phospho-Btk in OCT tumor sections and processed spleens

# OCT sections from MCL tumors were fixed with PFA on glass slides and permeabilized for 15 min with a solution of saponin 0.1% + FBS 10%, followed by a 30 min incubation with an Alexa488-labeled anti-Syk-phosphoTyr352, a PE-labeled anti-Btk-phosphoTyr223 (Becton Dickinson), or a Cy5-labeled anti-Lyn-phosphoTyr397 (Bioss Antibodies, Woburn, MA, USA) antibody. Coverslips were then mounted with DAPI-containing Fluoroshield mounting medium (Sigma-Aldrich) and visualized on a Nikon H5505 microscope by means of a 20X/1.30 NA oil objective (Nikon, Amsterdam, Netherlands) with the use of Isis Imaging System v5.3 software (MetaSystems GmbH).

# In the FL model, splenocytes were obtained by homogenizing harvested spleens and filtering through 70 μm nylon sieves (Becton Dickinson). Erythrocytes were lysed using ACK buffer (Quality Biological, Gaithersburg, MD, USA) and human B cells were labeled with anti-CD45-Pacific Blue (Thermo Fisher) antibody, followed by recounting on an Attune cytometer. In parallel, parts of the spleen homogenates were stained with an anti-CD45-Pacific Blue or an anti-CD20-PE (Beckman Coulter, L’Hospitalet de Llobregat, Spain) antibody, prior fixation, permeabilization and phospho-BCR kinase labeling as above, followed by analysis on either an Attune or a FACSCalibur (Becton Dickinson) cytometer.

# Generation of ibrutinib-resistant cells

# UPN-1 cells were initially treated for 96 hours with 1 µM ibrutinib and then were cultured in drug-free medium containing 20% FCS. After cell growth recovered, cells were treated with ibrutinib for an additional 72 hours, and the selection cycle was repeated at the same drug concentration until cell growth recoveries were obtained within 2 weeks. At this point drug concentration was increased to the next steps (2 µM, 5 µM, 7.5 µM, and, finally, 10 µM ibrutinib). After repeated rounds of selection with the 10 µM dose of ibrutinib over a period of 5 months, the established resistant cell line was designated “UPN-IbruR.” At this point, this cell line was cryopreserved and cultured in the presence of the Btk inhibitor.

# *BTK* and *PLCG2* sequencing

# DNA was extracted from UPN-IbruR cells using the QIAamp DNA Mini Kit (Qiagen; Venlo, Netherlands) in automated (QIAcube) extractions according to manufacturer’s instruction. We amplified by PCR the exon 15 of *BTK* (aa 450-522) and exon 18 of *PLCG2*. PCR products were treated with ExoSap IT (USB Corporation) and sequenced using ABI Prism BigDye terminator v3.1 (Applied Biosystems) using 5 pmol of each primer. Sequencing reactions were run on an ABI-3730 automated sequencer (Applied Biosystems). All sequences were examined with the Mutation Surveyor DNA Variant Analysis Software (Softgenetics).

# Pharmacokinetics evaluation of IQS019 in mouse

# IQS019-2MeSO_3_H PK study was performed at Crown Bioscience (Taicang, China). Briefly, IQS019-2MeSO_3_H was administered by p.o. at a single dose of 25 mg/kg to 3 male ICR mice. Plasma was recovered after 15 min, 30 min, 1h, 2h, 4h, 6h, 8h, and 24h and mixed with acetonitrile containing 200 ng/ml tolbutamide and 200 ng/ml propranolol. After a centrifuge step, supernatant was mixed with 0.1% formic acid and injected into an API 4000 mass spectrometer (Applied Biosystems). PK parameters were calculated using mean plasma concentration time data by Phoenix WinNonlin software v6.3, using a non-compartmental model.

# SUPPLEMENTAL TABLES

**Supplemental Table S1.- Dose-dependent inhibitory activity of IQS019 against TK and TKL kinase subfamilies**

| Kinase | Family | Kinase activity inhibition upon IQS019 exposure | |
| --- | --- | --- | --- |
|  |  | 0.1 μM | 10 μM |
| ABL1 E255K | TK | 22 | 83 |
| ABL1 F317I | TK | 19 | 80 |
| ABL1 G250E | TK | 12 | 74 |
| ABL1 H396P | TK | 50 | 94 |
| ABL1 M351T | TK | 43 | 94 |
| ABL1 Q252H | TK | 52 | 94 |
| ABL1 T315I | TK | 2 | 38 |
| ABL1 wt | TK | 42 | 93 |
| ABL1 Y253F | TK | 46 | 93 |
| ABL2 | TK | 74 | 100 |
| ACK1 | TK | 41 | 100 |
| ALK C1156Y (GST-HIS-tag) | TK | 4 | 88 |
| ALK F1174L (GST-HIS-tag) | TK | 0 | 80 |
| ALK F1174S (GST-HIS-tag) | TK | 0 | 74 |
| ALK L1196M (GST-HIS-tag) | TK | 1 | 92 |
| ALK R1275Q (GST-HIS-tag) | TK | 0 | 84 |
| ALK wt (GST-HIS-tag) | TK | 0 | 88 |
| AXL | TK | 5 | 66 |
| BLK | TK | 34 | 99 |
| BMX | TK | 34 | 99 |
| BRK | TK | 97 | 117 |
| BTK | TK | 19 | 94 |
| CSF1-R | TK | 19 | 96 |
| CSK | TK | 30 | 99 |
| DDR2 N456S | TK | 97 | 95 |
| DDR2 T654M | TK | 20 | 74 |
| DDR2 wt | TK | 96 | 96 |
| EGF-R d746-750 | TK | 84 | 99 |
| EGF-R d747-749/A750P | TK | 71 | 99 |
| EGF-R d747-752/P753S | TK | 78 | 99 |
| EGF-R d752-759 | TK | 69 | 99 |
| EGF-R G719C | TK | 91 | 100 |
| EGF-R G719S | TK | 82 | 100 |
| EGF-R L858R | TK | 80 | 99 |
| EGF-R L861Q | TK | 87 | 100 |
| EGF-R T790M | TK | 24 | 94 |
| EGF-R T790M/L858R | TK | 29 | 96 |
| EGF-R wt | TK | 80 | 99 |
| EPHA1 | TK | 84 | 100 |
| EPHA2 | TK | 75 | 100 |
| EPHA3 | TK | 21 | 69 |
| EPHA4 | TK | 40 | 92 |
| EPHA5 | TK | 11 | 92 |
| EPHA6 | TK | 10 | 88 |
| EPHA7 | TK | 11 | 65 |
| EPHA8 | TK | 42 | 97 |
| EPHB1 | TK | 70 | 100 |
| EPHB2 | TK | 0 | 75 |
| EPHB3 | TK | 5 | 65 |
| EPHB4 | TK | 31 | 98 |
| ERBB2 | TK | 29 | 96 |
| ERBB4 | TK | 75 | 100 |
| FAK aa2-1052 | TK | 4 | 59 |
| FER | TK | 4 | 86 |
| FES | TK | 10 | 88 |
| FGF-R1 V561M | TK | 0 | 78 |
| FGF-R1 wt | TK | 5 | 71 |
| FGF-R2 | TK | 5 | 75 |
| FGF-R3 G697C | TK | 7 | 86 |
| FGF-R3 K650E | TK | 1 | 81 |
| FGF-R3 K650M | TK | 9 | 84 |
| FGF-R3 wt | TK | 0 | 86 |
| FGF-R4 | TK | 0 | 35 |
| FGR | TK | 76 | 100 |
| FLT3 D835Y | TK | 1 | 22 |
| FLT3 ITD | TK | 4 | 27 |
| FLT3 wt | TK | 5 | 25 |
| FRK | TK | 61 | 99 |
| FYN | TK | 49 | 98 |
| HCK | TK | 50 | 99 |
| IGF1-R | TK | 0 | 67 |
| INS-R | TK | 0 | 37 |
| INSR-R | TK | 5 | 31 |
| ITK | TK | 0 | 79 |
| JAK1 | TK | 7 | 9 |
| JAK2 | TK | 9 | 43 |
| JAK3 | TK | 15 | 39 |
| KIT A829P | TK | 2 | 79 |
| KIT D816H | TK | 21 | 87 |
| KIT D816V | TK | 23 | 90 |
| KIT T670I | TK | 0 | 57 |
| KIT V559D | TK | 4 | 85 |
| KIT V559D/T670I | TK | 0 | 63 |
| KIT V559D/V654A | TK | 0 | 71 |
| KIT V560G | TK | 12 | 92 |
| KIT V654A | TK | 1 | 64 |
| KIT wt | TK | 3 | 67 |
| LCK | TK | 81 | 99 |
| LTK | TK | 1 | 88 |
| LYN | TK | 65 | 99 |
| MATK | TK | 0 | 55 |
| MERTK | TK | 0 | 86 |
| MET D1228H | TK | 7 | 34 |
| MET D1228N | TK | 7 | 39 |
| MET F1200I | TK | 0 | 16 |
| MET M1250T | TK | 0 | 29 |
| MET wt | TK | 0 | 39 |
| MET Y1230A | TK | 6 | 28 |
| MET Y1230C | TK | 8 | 40 |
| MET Y1230D | TK | 0 | 23 |
| MET Y1230H | TK | 0 | 21 |
| MET Y1235D | TK | 0 | 86 |
| MUSK | TK | 14 | 32 |
| NMP1ALK | TK | 0 | 76 |
| NMP1ALK F1174L | TK | 0 | 56 |
| PDGFR-alpha D842V | TK | 19 | 76 |
| PDGFR-alpha T674I | TK | 20 | 31 |
| PDGFR-alpha V561D | TK | 27 | 94 |
| PDGFR-alpha wt | TK | 0 | 68 |
| PDGFR-beta | TK | 6 | 62 |
| PYK2 | TK | 0 | 62 |
| RET E762Q | TK | 2 | 87 |
| RET G691S | TK | 0 | 83 |
| RET M918T | TK | 1 | 86 |
| RET R749T | TK | 0 | 88 |
| RET R813Q | TK | 0 | 89 |
| RET S891A | TK | 9 | 94 |
| RET V804L | TK | 8 | 65 |
| RET V804M | TK | 0 | 65 |
| RET wt | TK | 0 | 46 |
| RET Y791F | TK | 5 | 89 |
| RON | TK | 10 | 97 |
| ROS | TK | 1 | 20 |
| SRC (GST-HIS-tag) | TK | 59 | 99 |
| SRMS | TK | 1 | 65 |
| SYK aa1-635 | TK | 17 | 90 |
| TEC | TK | 0 | 65 |
| TIE2 R849W | TK | 0 | 86 |
| TIE2 wt | TK | 0 | 90 |
| TIE2 Y1108F | TK | 10 | 87 |
| TIE2 Y897S | TK | 8 | 92 |
| TRK-A | TK | 3 | 72 |
| TRK-B | TK | 0 | 73 |
| TRK-C | TK | 0 | 69 |
| TXK | TK | 18 | 78 |
| TYK2 | TK | 8 | 37 |
| TYRO3 | TK | 29 | 94 |
| VEGF-R1 | TK | 1 | 54 |
| VEGF-R2 | TK | 0 | 37 |
| VEGF-R3 | TK | 4 | 28 |
| YES | TK | 61 | 100 |
| ZAP70 | TK | 12 | 66 |
| ACV-R1 | TKL | 45 | 83 |
| ACV-R1B | TKL | 37 | 82 |
| ACV-R2A | TKL | 64 | 94 |
| ACV-R2B | TKL | 31 | 88 |
| ACV-RL1 | TKL | 62 | 99 |
| BMPR1A | TKL | 0 | 49 |
| B-RAF V600E | TKL | 57 | 93 |
| B-RAF wt | TKL | 62 | 97 |
| IRAK1 | TKL | 10 | 52 |
| IRAK4 (untagged) | TKL | 10 | 86 |
| LIMK1 | TKL | 0 | 1 |
| LIMK2 | TKL | 0 | 10 |
| LRRK2 G2019S | TKL | 6 | 51 |
| LRRK2 I2020T | TKL | 0 | 0 |
| LRRK2 R1441C | TKL | 1 | 0 |
| LRRK2 wt | TKL | 0 | 0 |
| MLK4 | TKL | 9 | 34 |
| RAF1 Y340D/Y341D (untagged) | TKL | 55 | 90 |
| RIPK2 | TKL | 99 | 92 |
| RIPK5 | TKL | 3 | 24 |
| TGFB-R1 | TKL | 5 | 84 |
| TGFB-R2 | TKL | 12 | 68 |
| ZAK | TKL | 0 | 47 |

# Supplemental Table S2.- Characteristics of CLL patients

| Patients | Gender^a^ | Age at diagnosis (years) | Cellsource^b^ | Tumoralcells (%)^c^ | *IGHV*status^d^ | *TP53* status^e^ | Cytogeneticalterations |
| --- | --- | --- | --- | --- | --- | --- | --- |
| CLL n.1 | F | 53 | PB | 95 | UM | wt | del(13q) |
| CLL n.2 | M | 64 | PB | 80 | M | wt | trisomy 12 |
| CLL n.3 | M | 58 | PB | 95 | M | wt | normal |
| CLL n.4 | M | 56 | PB | 79 | M | wt | n.d. |
| CLL n.5 | F | 52 | PB | 85 | M | wt | del(13q) |
| CLL n.6 | M | 63 | PB | 97 | UM | wt | del(13q) |
| CLL n.7 | M | 54 | PB | 92 | M | wt | del(13q) |
| CLL n.8 | M | 78 | PB | 94 | UM | wt | del(13q) |
| CLL n.9 | M | 67 | PB | 97 | UM | wt | normal |
| CLL n.10 | M | 44 | PB | 97 | UM | wt | del(13q) |
| CLL n.11 | M | 66 | PB | 96 | UM | wt | (11q)del |
| CLL n.12 | F | 49 | PB | 94 | M | wt | del(13q) |
| CLL n.13 | F | 73 | PB | 94 | UM | wt | trisomy 12 |

# ^a^ F: female; M: male

# ^b^ PB: peripheral blood; LN: lymph node

# ^c^ CD19+ tumor cells determined by flow cytometry

# ^d^ *IGHV* mutational status was done according to European Research Initiative on CLL (ERIC) guidelines ^42^.

# ^e^ 17p13 deletion was assessed by fluorescence in situ hybridization and *TP53* mutational status was analyzed by direct sequencing

UM indicates unmutated; M, mutated; wt, wild type; n.d., not determined; del, deletion; dup, duplication; add, addition.

**Supplemental Table S3.- PK parameters and plasma concentration of IQS019-2MeSO3H *vs* ibrutinib**

| Parameter | IQS019 | Ibrutinib ^a and ref [42]^ |
| --- | --- | --- |
| **t_1/2_ (h)** | 3.27 | 3.1 |
| **T_max_ (h)** | 4.0 | < 2.0 |
| **C_max_ (µM)** | 2 | 1.07 |
| **AUC_0-∞_ (h x ng/ml)** | 6966 | 568 |
| **Oral bioavailability** | 67% | 2-4% |

^a^[http://www.pharmacodia.com/yaodu/html/v1/chemicals/f15eda31a2da646eea513b0f81a5414d.html#pharmacokinetics1](http://www.pharmacodia.com/yaodu/html/v1/chemicals/f15eda31a2da646eea513b0f81a5414d.html#pharmacokinetics1)

**Supplemental figure legends**

**Figure S1. IQS019 tyrosine kinase inhibitory profiling.** Tyrosine kinase (TK) and tyrosine kinase-like (TKL) kinome tree was elaborated on the basis of residual *in vitro* kinase activity upon exposure to 100 nM or 1 μM IQS019, by means of Kinome Render software (<http://bcb.med.usherbrooke.ca/kinomerender.php>).

**Figure S2. Sensitivity of CLL primary cases to IQS019 is independent of *IGHV* mutational status and involves a caspase-dependent cell death process. (a)** CLL primary cells, 9 of them with ummutated (UM) and 6 with mutated (M) *IGHV* gene, were treated with increasing concentrations of IQS019 for 24h. Cell viability was determined by MTT method. Shown are the median values from each CLL group (UM and M), referred to control, untreated cells. **(b)** IQS019 induces caspase-dependent cell death in MCL (UPN-1) and in FL (DOHH-2) cell lines, as well as in two representative CLL primary cultures. Cells were exposed for 24 hours to 5 μM IQS019, in the presence of absence of the pan-caspase inhibitor Q-VD-OPh (10 μM). Apoptosis was determined by simultaneous cytofluorimetric detection of Annexin-V and caspase-3/7 activity. **(c)** A set of 6 CLL primary cultures were treated with IQS019 as indicated, followed by Western Blot detection of phospho-histone H3 (p-H3), using β- actin as a loading control.

**Figure S3. Flow cytometry determination of CXCR4 membrane expression in B-NHL cell lines.** Four representative cell lines were stained with a PE-labeled anti-CXCR4 antibody and analyzed on an Attune cytometer. CXCR4-specific signal (black curves) and isotypic control (grey filled curve) are represented.

**Figure S4. Safety and PK properties of IQS019-2MeSO_3_H in mice. (a)** Twenty SCID mice (10 males and 10 females) received a single intravenous injection of IQS019-2MeSO_3_H at a 2 mg/kg, 10 mg/kg, or 50 mg/kg dose, or equivalent volume of vehicle, and animal weight was recorded at days 1, 3, 4, 7, 11, 14, 18 and 21 post-treatment. **(b)** Mean plasma concentration of IQS019-2MeSO_3_H in ICR mice over the time, after a single p.o. administration of a 25 mg/kg dose of the compound.

# Figure S5. Comparison of parental and ibrutinib-resistant derived B-NHL cell line. (a) Dose-response of the UPN-1 parental, and UPN-IbruR derived cell line exposed for 72 hours to increasing concentrations of ibrutinib or IQS019. (b) BTK and PLCG2 exon sequencing in UPN-IbruR cells. (c) Western blot detection of the alternative NF-kappaB pathway component, p52, in UPN-1 and UPN-IbruR cells. β-actin was used as a loading control.

#

#

#

#

# 
